# Temporal radiographic and histological study of necrosis development in a mouse glioblastoma model

**DOI:** 10.3389/fonc.2022.993649

**Published:** 2022-10-14

**Authors:** Patricia P. Yee, Jianli Wang, Stephen Y. Chih, Dawit G. Aregawi, Michael J. Glantz, Brad E. Zacharia, Krishnamoorthy Thamburaj, Wei Li

**Affiliations:** ^1^ Division of Hematology and Oncology, Department of Pediatrics, Penn State College of Medicine, Hershey, PA, United States; ^2^ Medical Scientist Training Program, Penn State College of Medicine, Hershey, PA, United States; ^3^ Department of Radiology, Penn State College of Medicine, Hershey, PA, United States; ^4^ Neuro-Oncology Program, Department of Neurosurgery, Penn State College of Medicine, Hershey, PA, United States; ^5^ Penn State Cancer Institute, Penn State College of Medicine, Hershey, PA, United States; ^6^ Department of Neurology, Penn State College of Medicine, Hershey, PA, United States; ^7^ Department of Medicine, Penn State College of Medicine, Hershey, PA, United States; ^8^ Department of Biochemistry and Molecular Biology, Penn State College of Medicine, Hershey, PA, United States

**Keywords:** Glioblastoma, tumor necrosis, magnetic resonance imaging, MRI, mouse model, TAZ

## Abstract

Tumor necrosis is a poor prognostic marker in glioblastoma (GBM) and a variety of other solid cancers. Accumulating evidence supports that necrosis could facilitate tumor progression and resistance to therapeutics. GBM necrosis is typically first detected by magnetic resonance imaging (MRI), after prominent necrosis has already formed. Therefore, radiological appearances of early necrosis formation and the temporal-spatial development of necrosis alongside tumor progression remain poorly understood. This knowledge gap leads to a lack of reliable radiographic diagnostic/prognostic markers in early GBM progression to detect necrosis. Recently, we reported an orthotopic xenograft GBM murine model driven by hyperactivation of the Hippo pathway transcriptional coactivator with PDZ-binding motif (TAZ) which recapitulates the extent of GBM necrosis seen among patients. In this study, we utilized this model to perform a temporal radiographic and histological study of necrosis development. We observed tumor tissue actively undergoing necrosis first appears more brightly enhancing in the early stages of progression in comparison to the rest of the tumor tissue. Later stages of tumor progression lead to loss of enhancement and unenhancing signals in the necrotic central portion of tumors on T1-weighted post-contrast MRI. This central unenhancing portion coincides with the radiographic and clinical definition of necrosis among GBM patients. Moreover, as necrosis evolves, two relatively more contrast-enhancing rims are observed in relationship to the solid enhancing tumor surrounding the central necrosis in the later stages. The outer more prominently enhancing rim at the tumor border probably represents the infiltrating tumor edge, and the inner enhancing rim at the peri-necrotic region may represent locally infiltrating immune cells. The associated inflammation at the peri-necrotic region was further confirmed by immunohistochemical study of the temporal development of tumor necrosis. Neutrophils appear to be the predominant immune cell population in this region as necrosis evolves. This study shows central, brightly enhancing areas associated with inflammation in the tumor microenvironment may represent an early indication of necrosis development in GBM.

## Introduction

Glioblastoma (GBM) is the most common and aggressive primary brain tumor in adults. GBM is almost always first captured on brain CT and/or MRI, with MRI as the current gold-standard radiologic diagnostic modality that assists with pre-operative planning. Earlier studies reported that MRI findings closely correlated with histological grade of diffuse astrocytic tumors, including high grade gliomas (1, 2). Moreover, certain MRI features, such as contrast enhancement, necrosis, edema, mass effect, and intra-tumoral hemorrhage, have been shown to correlate with poor prognosis and clinical outcomes (3–5). Yet, unless identified incidentally on brain imaging obtained for other purposes, most GBMs remain undetected and undiagnosed until the tumors have progressed to the extent that they cause symptoms, edema, and brain compression as demonstrated by mass effect (6). By that time, rapid tumor expansion may have irreversibly damaged the surrounding normal brain parenchyma. Microscopic infiltration is usually so extensive at the time of diagnosis that tumors are incompletely resectable, so even maximal surgical resection is non-curative (6). Furthermore, genotoxic stress exerted by the hypoxic/ischemic tumor microenvironment promotes tumor evolution and molecular heterogeneity, rendering therapeutics ineffective. It has been well-established that early surgical resection results in improved overall survival among patients with both low-grade and malignant gliomas (7, 8). Under the assumption that early tumor detection can lead to early surgical resection, which in turn improves the overall survival, it is imperative to identify diagnostic markers to detect malignant tumor progression in the early and asymptomatic stages (4, 6). One histopathological feature associated with GBM progression is the formation of a necrotic core, caused by large-scale cell/tissue death. Tumor necrosis is a poor prognostic marker in GBM and a variety of other solid cancers (9–11). Accumulating evidence suggests that a necrotic core may facilitate tumor progression and evolution, thus promoting acquisition of resistance and negatively impacting patients’ responses to therapeutics (12).

Clinically, GBM necrosis has been long used as a radiographic diagnostic criterion to differentiate glioblastoma from other, lower-grade gliomas (13), and it is typically first detected by MRI. However, since most patients do not undergo brain imaging until the later, symptomatic stages of tumor progression, prominent necrosis has already formed by the time tumors are detected on imaging and confirmed by histopathology (2, 13). Therefore, radiological appearances of early necrosis formation and the temporal-spatial development of necrosis alongside tumor progression remain poorly understood. This knowledge gap leads to a lack of reliable radiographic diagnostic/prognostic markers in early GBM progression to detect necrosis.

It is commonly thought that necrosis is due to chronic ischemia-linked oxygen and nutrient deprivation in cancers and that the resulting metabolic stress is the cause of cell death. At the cellular level, although necrosis was previously thought to be a catastrophic and disordered cell death process (14), studies in a variety of pathological situations have found that necrosis can occur in a regulated fashion and includes several cell death mechanisms (15, 16). Whether necrosis in cancers is regulated through similar mechanisms remains unclear. Recent studies in glioblastoma suggested that immune components, such as neutrophils, can lead to oxidative stress-induced tumor cell death, such as ferroptosis, thereby amplifying tumor necrosis (17). Increased expression of ferroptosis-promoting genes was detected in the GBM necrotic area (17). Ferroptosis-related genes were also linked to immunosuppressive microenvironment and poor prognosis of GBM (18–22). These studies suggested that ferroptosis is involved in the development of a necrotic core in GBM.

Recently, we reported a xenograft GBM murine model in which ectopic expression of an active Hippo pathway transcriptional coactivator with PDZ-binding motif (TAZ) mutant (TAZ^4SA^) in the LN229 human GBM cell line can lead to orthotopic tumors which recapitulate the extent of GBM necrosis seen among patients (17). In this study, we utilized this model to perform a temporal radiographic and histological study of necrosis development. Our study indicated that more prominently enhancing areas associated with inflammation in the tumor microenvironment may represent an early indication of necrosis development in GBM.

## Materials and methods

### Real-time brain MRI imaging on GBM tumor-bearing mice

**Figure 1:**
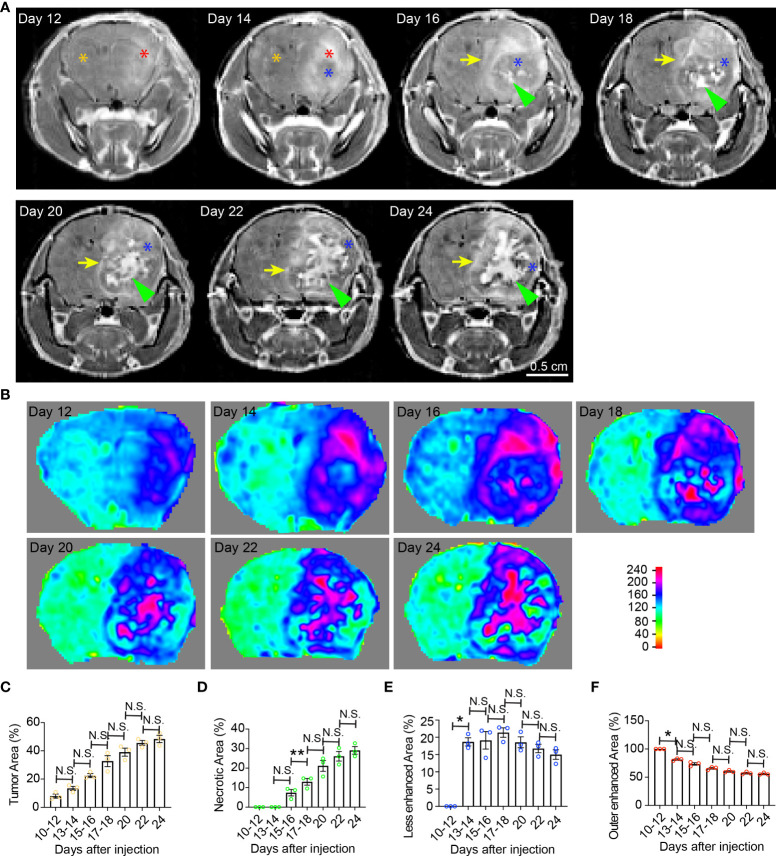
Temporal radiographic characterization of tumor necrosis development during early tumor progression *via* MR imaging. **(A)** Temporal development of GBM tumor necrosis during early, asymptomatic stages of tumor progression *via* representative serial T1-weighted post-gadolinium MRI scans of one LN229TAZ(4SA) tumor-bearing mouse. Orange asterisk: Normal brain parenchyma in the non-tumor containing hemisphere; red asterisk: Enhancing areas in the tumor-containing hemisphere; blue asterisk: The center of the tumor becomes less enhancing from day 14 onward, likely representing densely packed tumor cells; green arrowheads: Prominently enhancing foci within the less enhancing tumor stroma, likely representing active development of necrosis, which starts appearing from day 16. Yellow arrows: Peripheral enhancing interface between the less enhancing tumor tissue, as marked by blue asterisks, and the normal brain parenchyma. **(B)** Heatmaps of signal intensities generated using T1 post-contrast MRI images as in panel **(A)**. **(C)** Quantification of tumor area (outlined by the peripheral enhancing interface indicated by yellow arrows) normalized to whole brain area at each timepoint indicated above in panel **(A)**. p_ANOVA_=0.003. Results of post hoc test for each continuous time point are indicated. **(D)** Quantification of necrotic areas—labeled by green arrowheads in panel **(A)**—normalized to corresponding tumor at each timepoint indicated above. p_ANOVA_=0.0009. Results of post hoc test for each continuous time point were indicated. **(E)** Quantification of less-enhancing areas—labeled by blue asterisks in panel **(A)**—normalized to corresponding tumor at each timepoint indicated above. p_ANOVA_=0.012. Results of post hoc test for each continuous time point are indicated. **(F)** Quantification of the outer enhanced areas—labeled by red asterisks in panel **(A)**—normalized to corresponding tumor at each timepoint indicated above. p_ANOVA_=0.0011. Results of post hoc test for each continuous time point are indicated. Three mice were imaged as replicates with consistent observations; each datapoint shown in the bar graphs represents an animal (n=3). RM one-way ANOVA. Sidak’s multiple comparisons test was used in the post hoc test. All center values shown are mean values, and all error bars represent standard errors of the means (s.e.m). N.S.,p > 0.05. *,p < 0.05; **,p <0.01.

**Figure 2:**
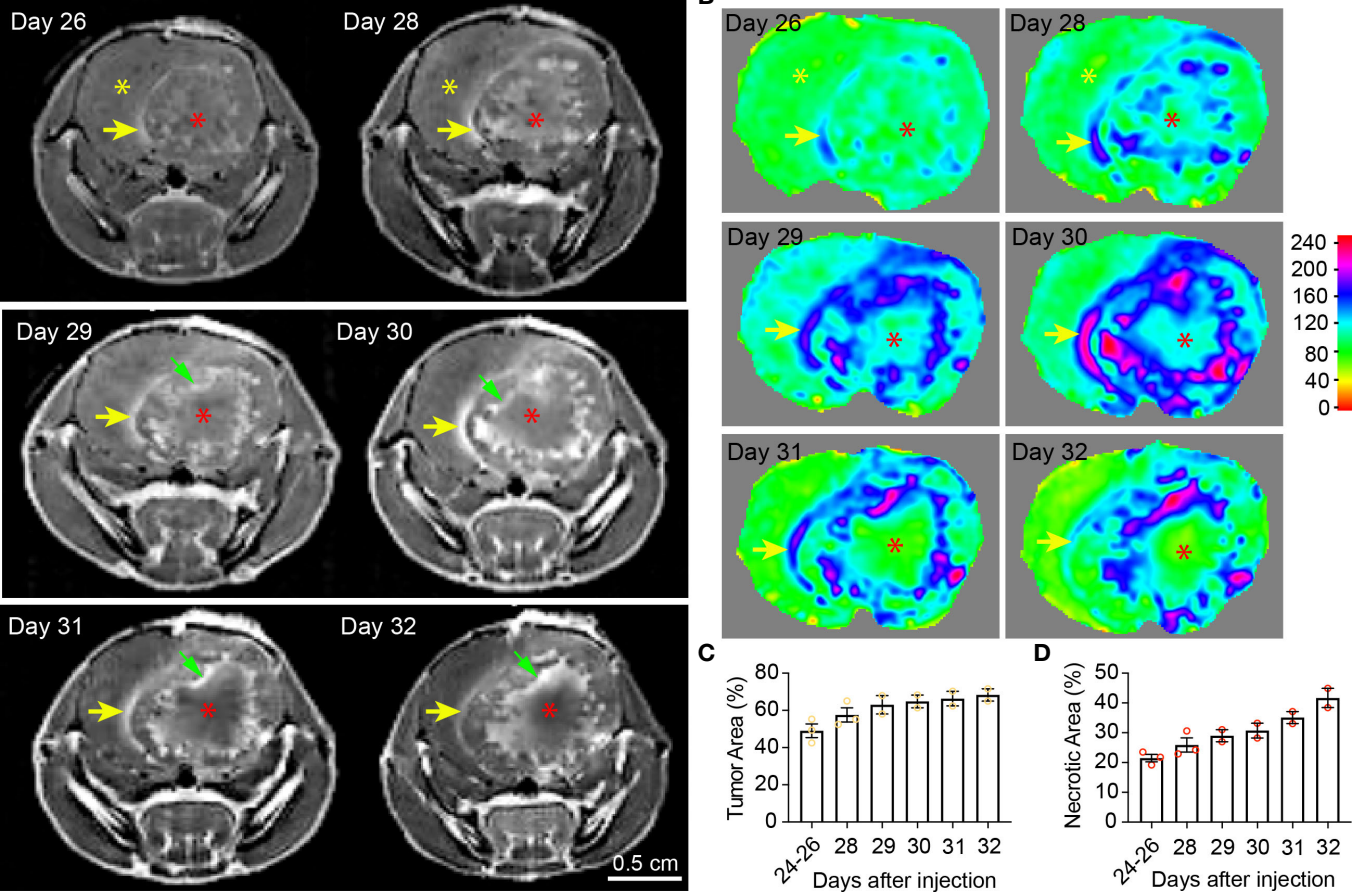
Temporal radiographic characterization of tumor necrosis development during late tumor progression *via* MR imaging. **(A)** Temporal development of GBM tumor necrosis during late tumor progression after onset of overt tumor-associated symptoms in tumor-bearing mice *via* representative serial T1-weighted post-gadolinium MRI scans of one LN229TAZ(4SA) tumor-bearing mouse (different from the animal used in **Figure 1**). Yellow asterisks: Brain parenchyma in the normal hemisphere; yellow arrows: More prominently enhancing rim at the outer edge of the tumor boundary, representing the tumor-infiltrating front; red asterisks: More prominently enhancing (but later unenhancing from day 29 onward) foci within the tumor stroma, representing active development of necrosis; green arrows: Prominently enhancing rim at the necrosis-cellular tumor (N-CT) interface, representing areas of active inflammation. **(B)** Heatmaps of signal intensities generated using T1 post-contrast MRI images as in panel **(A)**. **(C)** Quantification of tumor area (outlined by the peripheral enhancing interface indicated by yellow arrows) normalized to whole brain area at each timepoint indicated above. p=0.3684. **(D)** Quantification of necrotic areas—labeled by red asterisks as in panel **(A)**—normalized to corresponding tumor area at each indicated timepoint as above. p=0.1164. Three mice were imaged as replicates until reached the terminal stage (one at day 28, the other two at day 32; n=2-3); each datapoint shown in the bar graphs represents an animal. All center values shown are mean values, and all error bars represent standard errors of the means (s.e.m). Mixed-effects analysis.

**Figure 3:**
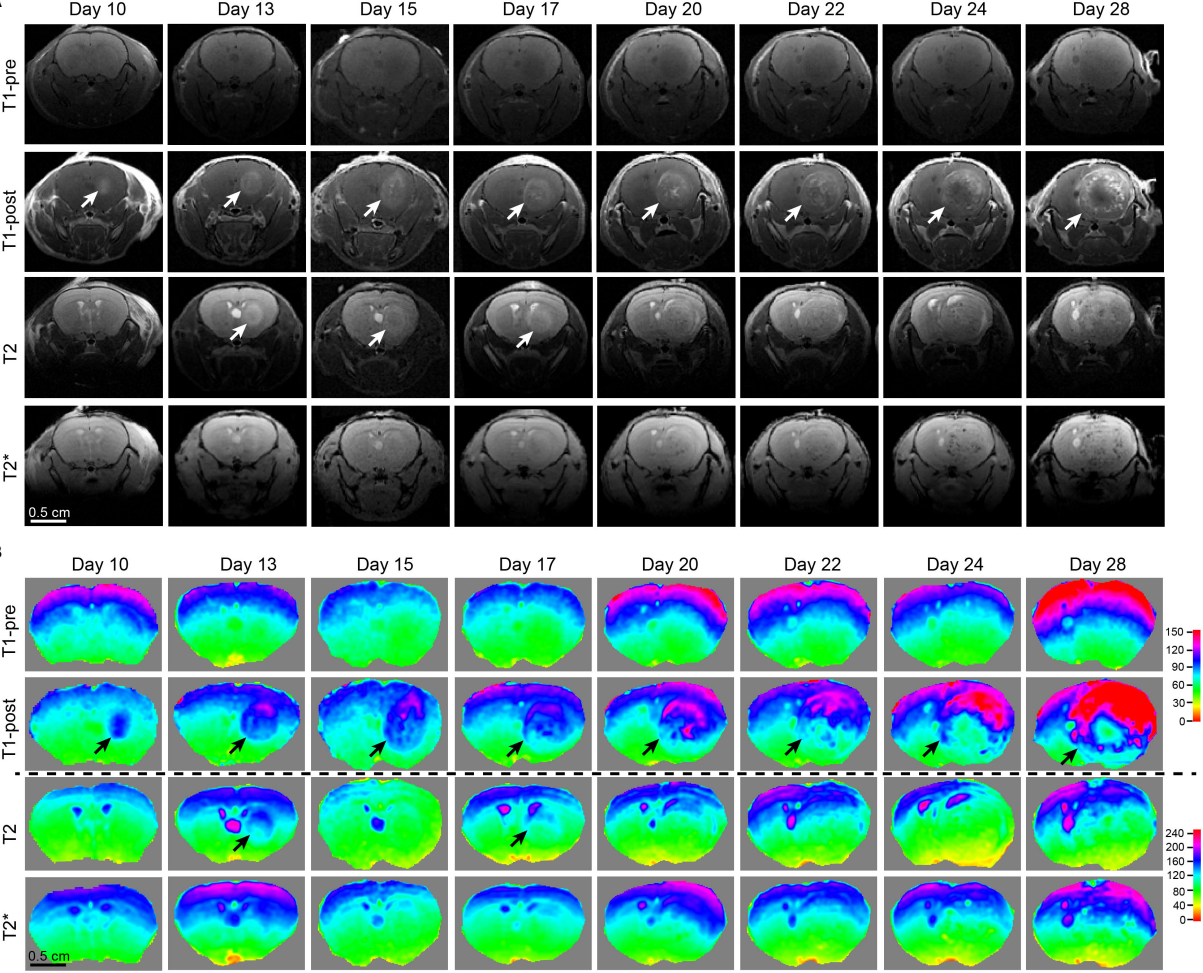
Comparison of different MRI modalities for temporal visualization of tumors and tumor necrosis using a murine GBM model. **(A)** Images acquired from various frequently utilized MR imaging modality for diagnostics and pre-operative planning in the clinical setting on the GBM tumor-bearing mouse devised by our lab. Starting from 10 days post tumor cell implantation, the mouse underwent serial brain MRI. Time points were pre-determined based on our previous histological studies on days 10, 13, 15, 17, 20, 22, 24, and 28, which is the end point of this GBM tumor-bearing mouse. **(B)** Heatmaps of signal intensities generated using MRI images as in panel **(A)**. Arrows: Enhancing areas likely representing tumors. One mouse was imaged.

MRI was conducted on a 7T MRI scanner (Bruker BIOSPEC 70/20 USR) with a 4-channel mouse brain surface array coil. Under anesthesia with 1.5-4% isoflurane, each animal was positioned prone on a 37°C heating pad with body temperature and respiratory rate monitored. The animal’s whole brain was imaged coronally in a spatial resolution of 133 μm × 133 μm × 500 μm using a T_1_-weighted spin-echo sequence (repetition time (TR)/echo time (TE)/flip angle (FA) = 500 ms/9.5 ms/90°), a T_2_-weighted rapid acquisition with relaxation enhancement sequence (TR/TE/FA = 2066 ms/36 ms/180°), and an 8-echo gradient-echo sequence for T_2_* mapping (TR/TE/FA = 1733 ms/4.5 ms/50°, echo spacing 5.5 ms). T_1_-weighted MRI was repeated about 15 minutes after a bolus injection of 0.2 mmol/kg gadolinium (Gadavist, Bayer Schering Pharma) through the lateral tail vein. The thickness for the mouse MRI images was 0.5 mm/slice. Heatmaps of MRI signal intensities at each timepoint indicated in **Figures 1B**, **2B**, **3B** were generated *via* ImageJ using the “Interactive 3D Surface Plot” function after manually outlining the brain area.

### Radiographic analysis of GBM necrosis *via* MRI

**Figure 4:**
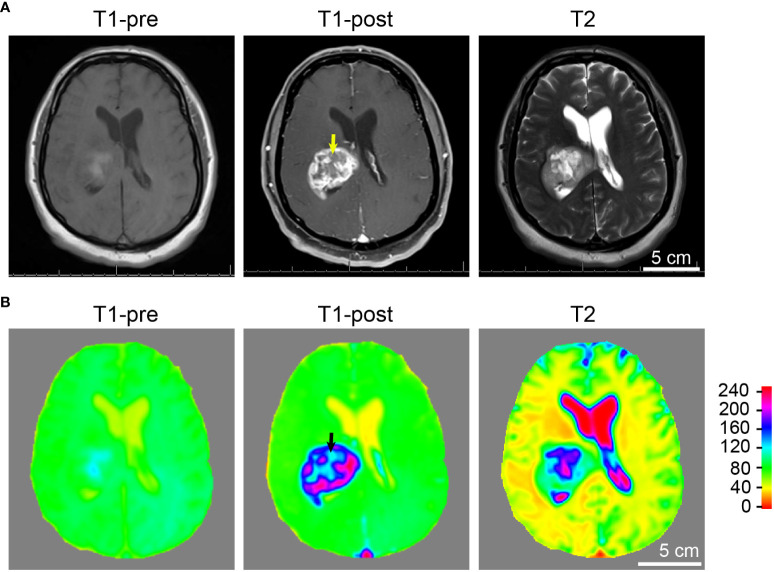
Tumor and necrosis visualized in a glioblastoma patient using standard MRI sequences. **(A)** A panel of representative images comparing T1 weighted pre-contrast (T1-pre), T1 weighted post-contrast (T1-post), and T2 sequences, which are routinely utilized in diagnostics and pre-operative planning for glioblastoma patient, are comparable to the images acquired using the murine GBM model which we devised. It is evident that T1-weighted post-gadolinium image remains by far the best sequence for the visualization of GBM tumor necrosis. **(B)** Heatmaps of signal intensities generated using MRI images as in panel **(A)**. Arrows: Less enhancing foci representing the central necrotic areas with irregular inner margins near the enhancing edges.

Subjects were retrospectively selected from a cohort of patients seen in Penn State Hershey Neuro-Oncology clinic between December 2018 and March 2019, and only patients with histopathologically confirmed WHO grade 4 malignant gliomas (i.e., GBMs) were included in this study (n=75). Pre-surgical, post-contrast axial T1-weighted fat saturated (T1 FS) MRI images with a slice thickness of 5 mm from patients with histologically-confirmed GBMs were retrospectively analyzed. MRI images were acquired *via* standard multi-contrast sequences including postcontrast fat saturated T1 TSE sequence using either 1.5T or 3.0T magnet (Siemens Healthcare) after injection of 0.1mmol/kg of gadolinium (Gadavist, Bayer Schering Pharma). Central necrosis was defined as non-enhancing areas within enhancing tumor with irregular inner margins on post-contrast T1-weighted images. Only pre-existing data were obtained *via* review of electronic medical records (EMR) and imaging studies (MRI), and therefore no further data collection or subject recruitment were conducted for this study. The study procedures and data collection were approved by the Institutional Review Board (IRB) of Penn State Hershey Medical Center. Per the Penn State IRB, human subject research presented in **Figure 4** was exempt from informed consent requirements. Heatmaps of signal intensities as in **Figure 4B** were generated *via* ImageJ using the “Interactive 3D Surface Plot” function after manually outlining the brain area.

### Mice and orthotopic xenograft tumor models

Six-to-eight-week-old female athymic nude mice (Nu(NCr)-Foxn1nu Strain Code: 490, Charles River) were used for the GBM orthotopic xenograft mouse models. For tumorigenesis experiments, human GBM cells were first transduced with a retroviral vector expressing firefly luciferase. These cells were then transduced with retroviral vectors expressing the indicated cDNAs. For each mouse, 3 × 10^5^ cells were injected into the right hemisphere at coordinates (+1, +2, -3). For tumor sample preparation and histology, whole brain tissue from tumor-bearing animals was fixed with 4% neutral-buffered formalin, embedded in paraffin, and submitted to the Penn State College of Medicine comparative medicine histology core, cut into sections 5 μm thick, and stained with hematoxylin and eosin (H&E). Areas of tumor and central necrosis were manually traced. All experiments described in this study were carried out with the approval of the Penn State University Institutional Animal Care and Use Committee and in accordance with its guidelines.

### Cells

Human GBM cell line, LN229 (CRL-2611), purchased from ATCC, was cultured in Dulbecco’s modified Eagle’s medium (DMEM; 10-013-CV, Corning) supplemented with 10% fetal bovine serum (FBS; Gibco, 10437028) and 1% Antibiotic–Antimycotic Solution (30-004-CI, Corning) at 37 °C with 5% CO_2_. The cell line was not authenticated in this study. The cell line was confirmed as *Mycoplasma* negative before experiments. Unless otherwise indicated, cells were grown to 50% confluence.

### Time course radiographic and histologic quantification of areas of interest (e.g., necrotic, peri-necrotic, cellular tumor, and total tumor)

**Figure 5:**
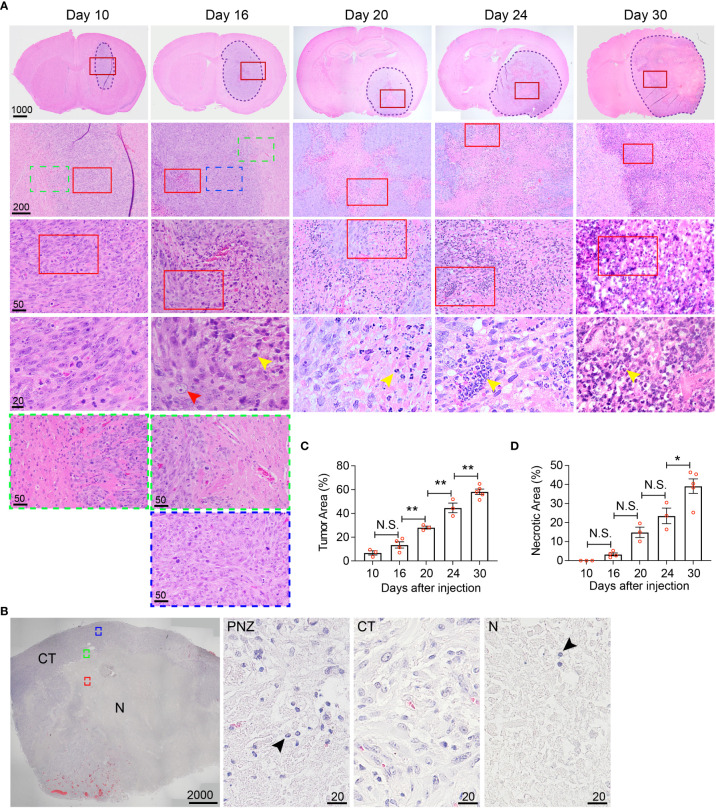
Histological characterization of the temporal development of GBM tumor necrosis. **(A)** Temporal development of GBM tumor necrosis from early to late tumor progression using representative time-course H&E-stained formaldehyde-fixed paraffin embedded sections of LN229TAZ(4SA) brain tumor-bearing mice. Each column represents images acquired from the same animal at a certain tumor progression stage. In each section, the tumor stroma has been traced out using a dashed line. Areas marked by red rectangles were further magnified in the images in the bottom of each column. For day 10 and 16 tumors, the areas marked by green (tumor border) or blue (cellular tumor) rectangles were also magnified and shown in the bottom of the correspondent columns. Yellow arrowheads: small, round, dark purple nuclei, likely representing mouse immune cells; red arrowheads: large, light purple nuclei, representing LN229 human tumor cells. **(B)** Representative image of a H&E-stained formaldehyde-fixed paraffin-embedded human GBM brain section showing cellular and necrotic tumor areas. The areas marked by green (peri-necrotic zone, PNZ), blue (cellular tumor, CT), or red (necrotic area, N) rectangles were magnified and shown on right. Arrowheads: small, round, dark purple nuclei, likely representing immune cells. Specimens from three different patients (n=3) were examined independently with similar observations. **(C)** Quantification of tumor area normalized to whole brain area at each timepoint indicated in panel **(A)**. p_ANOVA_< 0.0001. Results of post hoc test for each continuous time point are indicated. **(D)** Quantification of necrotic areas, appearing as pale pink, acellular regions, normalized to corresponding tumor area at each timepoint indicated above p_ANOVA_< 0.0001. Results of post hoc test for each continuous time point are indicated. Tumors from three to five tumor-bearing mice were sectioned and imaged in parallel as replicates with consistent observations (n=3-5); each datapoint shown in the bar graphs represents an animal. Scale bar is in µm. Ordinary one-way ANOVA. Sidak’s multiple comparisons test was used in the post hoc test. All center values shown are mean values, and all error bars represent standard errors of the mean (s.e.m). N.S., p > 0.05. *,p < 0.05; **,p < 0.01.

For quantification as in **Figures 1**, **2**, T1 post-gadolinium images were acquired as above. Quantification was performed using images containing tumors with the largest cross-sections. Each specific area of interest (e.g., yellow arrow and green arrowhead as in **Figure 1**) was manually traced using the freehand tool and measured using the “Analyze” function in ImageJ. Data for tumor areas were normalized to corresponding whole brain area, whereas other areas of interest (i.e., necrotic, enhanced, and less enhanced areas) were normalized to corresponding tumor size. For quantification shown in **Figure 5**, paraffin-embedded, H&E-stained sections collected at each indicated timepoint prepared as above were used for quantification. Necrosis (N) is defined as acellular regions (appearing pale pink) within tumors as identified by H&E stain, and a cellular tumor (CT) region is defined as a hypercellular region. Quantification was performed using sections with the largest cross-sections. Regions of interest were manually traced using the freehand tool and measured using the “Analyze” function in ImageJ. All statistical calculations and plotting were performed using GraphPad Prism 9.

### Immunofluorescent staining and analyses

**Figure 6:**
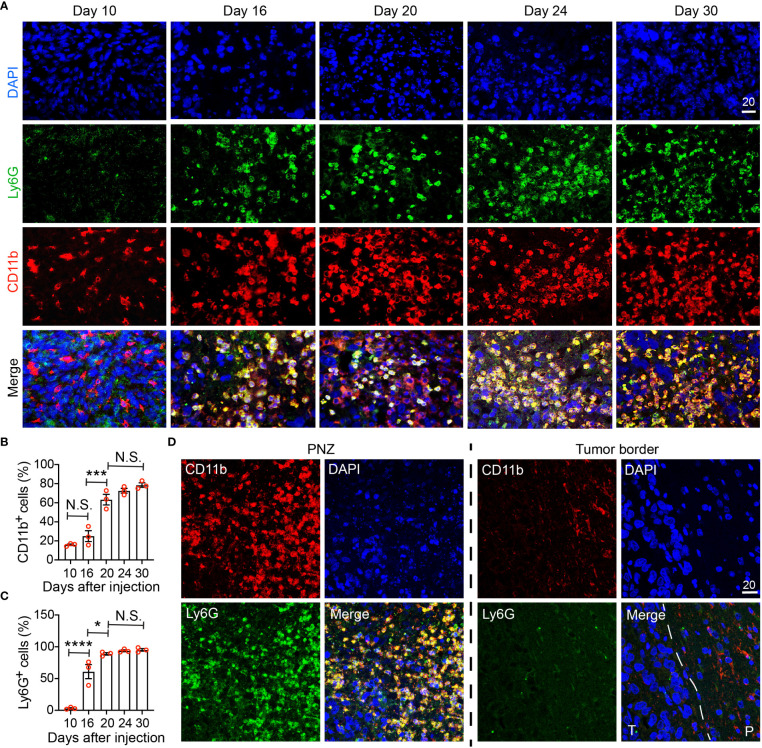
Immunohistochemical characterization of the temporal development of GBM tumor necrosis. **(A)** Temporal development of GBM tumor necrosis from the early to the late tumor progression using representative time-course CD11b, Ly6G, and DAPI immunofluorescence staining on brain sections collected from LN229^TAZ(4SA)^ brain tumor-bearing mice. (same cohort of animals as in **Figure 5**). For day 10, an area of cellular tumor was imaged. For day 16-30, an area of peri-necrotic zone at the interface between cellular and necrotic tumor was imaged for each sample. Images in each column were acquired from the same animal. **(B)** Percentage of CD11b^+^ cells normalized to all DAPI^+^ cells in one high-power 40X field collected at each timepoint indicated in panel **(A)**. p_ANOVA_<0.0001. Results of post hoc test for each continuous time point are indicated. **(C)** Percentage of Ly6G^+^ cells normalized to all CD11b^+^ cells in one high-power 40X field collected at each timepoint indicated in panel **(A)**. p_ANOVA_<0.0001. Results of post hoc test for each continuous time point are indicated. Each datapoint shown in the bar graphs represents an animal. Ordinary one-way ANOVA. Sidak’s multiple comparisons test was used in the post hoc test. All center values shown are mean values, and all error bars represent standard errors of the means (s.e.m). N.S., p > 0.05. *,p < 0.05; ***,p < 0.001; ****,p < 0.0001. **(D)** Comparison of immunofluorescent images as above acquired from a terminal-stage (i.e., day 30) section between the peri-necrotic zone, denoted by PNZ, and from the tumor border; dashed line outlines the tumor (T) and normal parenchyma (P) border. Tumors from three tumor-bearing mice were sectioned and imaged in parallel as replicates with consistent observations (n=3). Scale bar is in µm.

Immunofluorescent staining was performed as previously described (23). For histological samples, paraffin-embedded 5-μm sections were deparaffinized and rehydrated in successive baths of xylene and ethanol (100%, 95%, 70%, and 50%), followed by heat-induced (95 °C) epitope retrieval in 10 mM sodium citrate buffer (pH = 6.0). After one-hour block with 5% BSA/PBS at room temperature, samples were incubated overnight at 4°C with primary antibodies diluted in 2.5% BSA/0.05% Triton X-100/PBS. The next day, sections were washed three times with 0.1% Triton X100/PBS prior to incubation with secondary antibody diluted in 2.5% BSA/0.05% Triton X-100/PBS for 60-90 minutes at room temperature. Then, sections were again washed three times with 0.1% Triton X-100/PBS, labeled with 4,6-diamidino-2-phenylindole (DAPI) for nuclear visualization, rinsed with PBS, and mounted in ProLong Gold Antifade Mountant (P10144, Invitrogen). Primary antibodies, ultra-LEAF purified rat anti-mouse Ly-6G (1A8, 127620, Biolegend) and rabbit anti-mouse/human CD11b (ab133357, Abcam), were diluted at a 1-to-100 concentration. Secondary antibodies, Alexa Fluor 488 donkey anti-rat IgG (712-545-150, Jackson ImmunoResearch) and Alexa Fluor 594 donkey anti-rabbit IgG (711-585-152, Jackson ImmunoResearch), were diluted at 1-to-200 concentration. For analysis of percentage of CD11b+ and Ly6G+ doubly-positive cells in tumors collected at various time points as in **Figure 6**, images were acquired within 1–3 days following immunofluorescent staining as above using an Olympus CX41 microscope PLCN 40x objective. All images were taken within the tumor adjacent to the central necrosis, in so-called peri-necrotic regions, where infiltrating immune cells were most abundant. All images were first converted to 8-bit grayscale images, followed by background subtraction, thresholding, and quantification using the Analyze Particles function in ImageJ. Quantifications of double-positive and triple-positive cells were performed by using the same approach as above on images generated by Image Calculator using the “AND” function in ImageJ. Corresponding DAPI images were obtained for visualization of cellular nuclei and for normalization of percentage of positive signals per cell. Data were plotted as percentage of CD11b+ (singly-positive) normalized to all DAPI+ cells within one high-power 40X field, or percentage of CD11b+–Ly6G+ (doubly-positive) cells normalized to all CD11b+ cells within one high-power 40X field. For analysis of CD11b+ and Ly6G+ cells in tumors or tumor borders as in **Figure 6D**, images were acquired within 1–3 days following immunofluorescent staining as above using a Leica SP8 inverted confocal laser scanning microscope with 63x objective. Images were then stacked with maximal intensity using the “Z project” function and merged using ImageJ.

## Results

### Temporal radiographic characterization of tumor necrosis development *via* MR imaging

Clinically, GBM tumor necrosis is typically first identified radiographically *via* T1-weighted MRI with contrast prior to surgery, as histopathological examination cannot be performed until the tumor specimen has been removed. To study the development of tumor necrosis in GBM, we carried out a longitudinal radiographic imaging study on LN229^TAZ(4SA)^ tumor-bearing mice, in which tumor necrosis progressively forms during tumor development (17). LN229^TAZ(4SA)^ tumor-bearing mice were imaged every other day in the early tumor progression stages (starting 10-12 days after tumor implantation) before the onset of any overt tumor-associated symptoms (around 24 days after tumor implantation). During early tumor progression, neoplastic tissue can be readily distinguished from the normal brain tissue by its enhanced contrast signals (**Figures 1A, B**, day- 12 and 14 scans, red asterisks). The contrast enhancement is likely the result of leaky vasculature and the lack of a blood-brain barrier. In addition to neoplastic tissues with leaky vessels, tissues undergoing active inflammation can demonstrate enhancement due to infiltration of immune cells with resultant edema. We noticed the centers of tumors started showing less enhancement on day 14 (**Figure 1A**, blue asterisk; and **Figure 1B**). This likely represents proliferating tumor cells becoming more compact in the tumor center compared to the periphery. H&E staining of tissue sections at this stage confirmed this notion (**Figure 5**, day-10 scan). The decreased enhancement of solid tumor components between the contrast-enhancing rims may be related to the dense packing of tumor cells in that region, leaving little extracellular space and therefore resulting in reduced contrast enhancement. Changes in MRI appearance for necrosis were first detected on day-16 scan, in which the center of the tumor contained heterogeneous and brighter-enhancing signals on post-contrast T1 spin echo sequence (**Figure 1A**, day-16 scan, green arrowhead; and **Figure 1B**, day-16 scan), likely representing actively developing central necrosis, and a contrast-enhancing rim surrounding the tumor, likely representing tumor-infiltrating fronts (**Figure 1A**, day-16 scan, yellow arrow; and **Figure 1B**, day-16 scan). The relatively less-enhancing core (**Figure 1A**, day 16-24 scans, blue asterisks) of the tumor surrounding the brightly enhancing central necrosis (**Figure 1A**, day 16-24 scans, green arrowheads) was in turn surrounded by a relatively more enhancing peripheral rim (**Figure 1A**, day 16-24 scans, yellow arrows). Quantification of these radiological features showed that tumor and necrotic areas, which are the areas enclosed by the outer contrast-enhancing rim (**Figure 1A**, day 16-24 scans, yellow arrows) and the intratumoral contrast-enhancing foci (**Figure 1A**, day 16-24 scans, green arrowheads), respectively, both gradually enlarged with tumor growth (**Figures 1C, D**). In contrast, the relatively less-enhancing areas (**Figure 1A**, day 14-24 scans, blue asterisks) did not expand along with tumors after they appeared around day 14 (**Figure 1E**), while the peripherally-enhancing rims (**Figure 1A**, day- 12 and 14 scans, red asterisks) became thinner (**Figure 1F**).

In the second imaging study, MRI scans were acquired more frequently after the LN229^TAZ(4SA)^ tumor-bearing mice developed overt tumor-associated symptoms (i.e., starting 24 days after tumor implantation). At this time, the relatively more prominent contrast-enhancing rim on the outer edge of the tumor boundaries remained visible throughout the course of imaging (**Figures 2A, B**, yellow arrows). Using this contrast-enhancing rim as the border of the expanding tumor front, we found that tumors continue expanding (**Figure 1C**), although the expansion is slower when compared to earlier stages, between days 11-24 (comparing **Figure 2C** to **1C**). Notably, the regions representing central tumor necrosis, which initially demonstrated more prominent enhancement than other solid tumor areas (**Figures 2A, B**, day- 26, 28 and 29 scans, red asterisks vs. yellow asterisks), became gradually less contrast-enhancing from day 31 onward (**Figures 2A, B**, red asterisk on day-31 scan vs. red asterisks on day- 28 and 29 scans). The loss of contrast signal in the central areas reflects the occurrence of extensive tumor tissue death (**Figure 5A**, day-30 scan). We also observed another more brightly contrast-enhancing rim at the interface of solid tumor and central tumor necrosis (i.e., N-CT interfaces) (**Figure 2A**, green arrows) from day 29 onward after the implantation. This second rim of enhancement likely represented the active area undergoing inflammation flooded with infiltrating immune cells (e.g., tumor-associated neutrophils, TANs, and tumor-associated macrophages and microglia, TAMs) and interstitial fluid. Using this second contrast-enhancing rim as the outline of the necrotic area, we found that necrotic cores continue expanding at this stage (**Figure 1D**), although more slowly when compared to stages between days 11-24 (comparing **Figure 2D** to **1D**). Together, these temporal studies suggested hyperactivated TAZ-driven GBM tumor necrosis positively correlated with tumor and symptomatic progression, consistent with what has been reported in clinical human GBM studies. The solid portions of tumors between these two prominently enhancing rims were relatively less-enhancing (**Figures 2A, B**, day 26-32 scans) as observed in **Figure 1** (day 14-24 scans).

### Comparison of different MRI sequences for the temporal visualization of tumors and tumor necrosis

Tumor necrosis in GBM patients is typically first identified radiographically *via* T1-weighted MRI performed with a gadolinium contrast-enhancing agent. T1-weighted pre- and post-gadolinium images are especially useful in investigating breakdown of the blood-brain barrier (e.g., tumors, abscesses, brain inflammation, or viral encephalitis.) (24). In the imaging of GBM, T2-weighted sequences are almost always obtained concurrently given their capacity to detect tumor infiltration with edema. (T2*) or susceptibility weighted images are commonly utilized when attempting to detect structural changes related to intracranial hemorrhage (e.g., arteriovenous malformation, cavernoma, hemorrhage within a tumor, punctate hemorrhages in diffuse axonal injury, thrombosed aneurysm, or some forms of calcification.) (24, 25). To compare and contrast these different MRI modalities for the visualization of tumors and tumor necrosis in our GBM mouse model, we conducted a temporal radiographic imaging study on LN229^TAZ(4SA)^ tumor-bearing mice using different MRI modalities. When T1-weighted post-gadolinium imaging was utilized, temporal tumor growth and formation of the prominent necrotic core was visualized (**Figures 3A, B**, T1-post, arrows). Contrarily, in non-contrast T1 imaging, tumors were barely detectable (**Figures 3A, B**, T1-pre). While early-stage tumors were readily visible with enhanced signals in the T2 setting (day 13-17), they gradually lost their enhanced T2 signals and became almost isointense to the surrounding parenchyma in later stages (**Figures 3A, B**, T2, arrows). On T2* gradient echo images—specifically used for the detection of blood products or microhemorrhages in tumors (24, 25)– the tumors in the mouse model did not show enhanced signals (**Figures 3A, B**, T2*). To ensure the clinical translatability of observations from our GBM mouse model, we also obtained images acquired with different MRI sequences from GBM patients in the clinical setting. Similar to the above observation in our mouse model, tumor necrosis was best visualized in T1-weighted post-contrast images as less-enhancing areas with irregular inner margins near the enhancing edges (**Figures 4A, B**, T1-post, arrows). These results indicated that T1-weighted post-contrast MRI represents the best imaging modality for visualization and characterization of tumor necrosis both in pre-clinical and clinical GBM models, especially in advanced stages.

### Histological characterization of the temporal development of GBM tumor necrosis

While MRI studies provided real-time information about necrosis development with high clinical translatability, the resolution of biological/physiological occurrence provided by MRI scans is somewhat limited to the large-scale tissue level. To allow finer examination of necrosis development at the cellular level in the hyperactivated TAZ-driven mouse GBM model, we performed temporal histological studies by collecting brain tissue from the LN229^TAZ(4SA)^ tumor-bearing mice at different stages throughout tumor progression, from asymptomatic stages (i.e., on days 10, 16, and 20 after tumor implantation), to the symptomatic stage (i.e., from day 24 following implantation and onward), and eventually at the endpoint (i.e., day 30 after implantation). We saw that on day 10 after tumor implantation, tumor tissue was readily visible and could be distinguished from the normal brain parenchyma by its purple stain on H&E sections. At this time, tumors appeared relatively small and homogeneous, although tumor cells are more compact in the tumor center compared to the periphery (**Figure 5A**, day-10 sections, red-outlined vs. green-outlined areas). Consistent with above MRI studies, tumor sections collected from day 16 and onward after tumor implantation appeared more heterogeneous, with a small central eosinophilic, pale-pink appearing, acellular region of necrosis (**Figure 5A**, day-16 sections). At this stage, the tumor can be subdivided into three areas, including periphery (less dense, **Figure 5A**, day-16, green-outlined area), cellular tumor zone (dense, **Figure 5A**, day-16 sections, blue-outlined area), and necrotic area (**Figure 5**, day-16 sections, red-outlined area). These three areas correspond to the relatively more enhancing peripheral rim (**Figure 1A**, day 16-24 sections, yellow arrows), the relatively less enhancing core (**Figure 1A**, day 16-24 sections, blue asterisks) of the tumor, and the brightly enhancing central necrosis (**Figure 1A**, day 16-24 sections, green arrowheads), respectively, that were observed in the MRI scans. The histologically-distinct areas, including cellular tumor zone and necrotic area, can be similarly observed in the H&E sections obtained from a GBM patient (**Figure 5B**). Furthermore, in concordance with results from the above MRI studies, the size of tumor necrosis positively correlated with the size of LN229^TAZ(4SA)^ tumors and symptomatic progression in mice (**Figures 5C, D**). Interestingly, we consistently observed small round cells with dense, dark purple nuclei (yellow arrowheads), characteristic of mouse immune cells and morphologically distinct from human tumor cells with diffuse, light-purple nuclei (red arrowheads) in the peri-necrotic regions starting on day 16 after tumor implantation (**Figure 5A**), indicating that prominent infiltration of mouse immune cells began around this time. Morphologically-similar cells were also seen in the peri-necrotic zone (PNZ) and necrotic area of the samples from GBM patients (**Figure 5B**, arrowheads).

### Immunohistochemical characterization of the temporal development of GBM tumor necrosis

To validate our MRI results and to further examine the correlation between immune cells and tumor necrosis at various stages of tumor development, we performed immunohistochemistry using a commonly used myeloid cell marker, CD11b. Because neutrophils (i.e., tumor-associated neutrophils, TANs) have been reported to be enriched in the necrotic region (17), we also used Ly6G, a murine neutrophil marker, to monitor TANs. Brain tumors were visible on hematoxylin and eosin (H&E) staining at day 10 after tumor cell implantation, but there was no detectable tumor necrosis (**Figure 5A**, day-10 sections). In these tumors, CD11b^+^ cells could be detected, whereas few Ly6G^+^ cells were seen (**Figure 6A**, day-10 sections; **Figures 6B, C**). At day 16 after tumor cell implantation, the derived tumors contained a few necrotic foci that were infiltrated with cells labeled by both CD11b and Ly6G (**Figure 6A**, day-16 sections; **Figures 6B, C**). As tumors further progressed and the necrotic areas further expanded, CD11b^+^ cells were more frequently observed in the PNZ than in the tumor border, where few CD11b^+^ cells were found in the brain parenchyma (**Figure 6D**). As the CD11b^+^ cell population increased in the PNZ, the abundance of CD11b^+^Ly6G^+^ double-positive cells also increased (**Figures 6B, C**). These results indicated that CD11b^+^Ly6G^+^ cells spatially and temporally coincided with tumor necrosis and tumor progression in LN229^TAZ(4SA)^ tumors, consistent with what has been previously described (17). Overall, these results confirmed the above MRI observations that immune cells and tumor necrosis are temporally and spatially correlated.

## Discussion

While necrosis is common in solid malignancies at advanced stages, especially GBMs, the nature and mechanisms driving its development and evolution remain obscure, particularly in the early stages of tumor progression. Clinically, brain MRI plays a pivotal role and remains the primary follow-up modality in assessing therapeutic response and prognosis in GBM patients once the histopathological diagnosis has been confirmed. GBM patients typically undergo brain MRI once every 2-3 months following the initial tumor resection. While tumor necrosis is typically first detected *via* brain MRI among GBM patients, the radiological appearance and temporal evolution of early necrosis formation alongside tumor progression remains poorly understood, resulting in the lack of reliable radiographic diagnostic and prognostic markers in early GBM progression. This is unsurprising given that unless the lesion is identified incidentally, most GBM patients do not undergo brain imaging until they progress to the later, symptomatic stages when symptoms result from extensive tumor expansion and perilesional edema which compress normal brain parenchyma, distort vascular supply, and reconfigure the neurotransmitter environment. By such a time, prominent central tumor necrosis is already well established and easily detected on imaging.

In this study, we utilized an orthotopic xenograft GBM murine model which recapitulates the extent of GBM necrosis seen among patients (17) to fill the above gap in understanding *via* temporal radiographic and histological characterization of necrosis evolution, hoping to identify possible radiographic diagnostic/prognostic markers in early GBM progression. Overall, images acquired *via* T1- and T2- weighted (including T2*) MRIs on our murine model were comparable to those from GBM patients. Nevertheless, unlike intra-lesional blood products including microhemorrhages commonly reported in MRI images of GBM, we did not observe much susceptibility signals in our murine GBM tumor-bearing animals on T2* MRI, suggesting lack of intra-lesional blood products or microhemorrhages in our GBM murine model. This was unsurprising, given that we did not observe extensive intra-tumoral microvascular proliferation or subsequent hemorrhaging on histological studies with our murine GBM model. Additionally, the development of radiographically visualized necrosis positively correlates with the tumor size as well as symptomatic progression. Necrosis at later stages of tumor progression appears as a non-enhancing central-tumoral region on post-contrast T1-weighted images, probably due to extensive tumoral tissue death resulting in the loss of contrast enhancement. Such lack of central enhancement coincides with the radiographic and clinical definition of necrosis among GBM patients. Interestingly, while necrosis eventually loses enhancement and appears unenhancing on T1-weighted post-contrast MRI when compared to normal parenchyma, tissue actively undergoing necrosis first appears more prominently enhancing in the early stages of tumor progression. This feature probably coincides with active inflammation with resulting loss of the blood-brain barrier and extravasation of contrast. Moreover, as necrosis evolves, two contrast-enhancing rims are observed at later stages. The outer rim at the tumor border likely represents the infiltrating tumor edge, and the inner rim at the peri-necrotic region may represent locally infiltrating immune cells which facilitate the development of tumor necrosis, as reported in previous studies (17). These radiographic findings associated with early stages of GBM tumor progression and necrosis evolution, to our knowledge, have not yet been reported in literature.

There are several limitations to this study. First, findings reported in this study are limited to one type of murine xenograft model; whether these findings can be reproduced using other types of GBM murine xenograft models—in particular, GBM patient-derived xenograft murine models—and other non-xenograft models awaits future study. Second, the resolution of biological and physiological processes provided by MRI imaging is somewhat limited to the large-scale tissue level, and it would be clinically inappropriate to generate diagnoses or prognostic information regarding malignancy based solely on radiographic changes without verification from histological or molecular studies. This may be addressed in future murine studies by incorporating MRI compatible cellular contrast dye specific for tumor tissue as well as other tumor-associated immune cells (e.g., TANs or TAMs) as technology and tools in cellular and cell-tracking MRI evolve and become available (26–28).

It is well-established that both chemotherapy and radiation result in tumor tissue death, and therefore necrosis may be a useful predictor of tumor response to treatments. If we have a better understanding of the temporal evolution of intratumoral necrosis, and if we are able to accurately measure it, this may serve as a potential biomarker for monitoring treatment responses of individual tumors and allow tailoring of treatment in real time. Radiographically, the combination of chemotherapy and radiation often provokes contrast enhancement and seeming enlargement of the residual tumor, mimicking tumor progression in so-called pseudoprogression (29). It has long been a challenge to distinguish treatment-related necrosis and its resultant radiographic changes from true disease progression, especially among patients with treatment-resistant tumors. This poses a major hurdle in the follow-up and surveillance of patients with high-grade gliomas, including GBM, as additional surgical biopsy or multi-modal imaging studies, such as perfusion MRI or MR spectroscopy, are necessary for a conclusive diagnosis, which risks delaying treatment of true disease progression. Conventional grading of gliomas does not predict therapeutic response of individual tumors even with same histological grade, and as a result, contrast-enhanced MRI has been the most widely utilized clinical tool to guide diagnosis, surgical navigation, and radiation treatment planning. Additionally, it is the most common objective assessment with which to monitor treatment responses to standard adjuvant chemotherapy and radiation. Moreover, for patients with gliomas that are non-resectable due to being in eloquent locations, MRI remains the gold standard for routine surveillance of these patients and monitoring of potential malignant transformation from low- to high-grade tumors. In some circumstances, MRI may be the only assessment used for diagnosis and for differentiation of low-grade tumors from high-grade ones.

Therefore, knowledge gained from our study may provide neuroradiologists with new insights to more accurately interpret post-treatment radiographic changes among GBM patients, allowing recognition of radiographic changes of necrosis and low-to-high grade malignant transformation at earlier stages, which could in turn facilitate and guide treatment planning among a multi-disciplinary neuro-oncology team to minimize delays and improve success. These potential implications remain to be further explored in future studies.

## Data availability statement

The original contributions presented in the study are included in the article/supplementary material. Further inquiries can be directed to the corresponding author.

## Ethics statement

The studies involving human participants were reviewed and approved by Penn State University Institutional Review Board. Written informed consent for participation was not required for this study in accordance with the national legislation and the institutional requirements. The animal study was reviewed and approved by Penn State University Institutional Animal Care and Use Committee.

## Author contributions

PY and WL conceived the project and designed the experiments. PY and WL performed stereotaxic intracranial surgeries in mice. PY performed histological studies of mouse brain tumor specimens with assistance from SC and WL. JW performed mouse MRI. PY performed mouse and human radiographical analyses under the supervision of KT and WL. DA, MG, BZ, and KT provided MRI results from GBM patients seen at the Neurooncology clinic at Penn State Hershey Medical Center. PY and WL wrote an original manuscript. All authors provided intellectual input and edited the manuscript. WL supervised all aspects of the work.

## Funding

We acknowledge support from the National Institutes of Neurological Disorders and Stroke (R01 NS109147 and NS119547 to WL), Penn State College of Medicine Medical Scientist Training Program (5T32GM118294 to PY through PSU), and the Four Diamonds (to PSU).

## Acknowledgments

We would like to thank Dr. Kun-liang Guan for reagents, members of the Li Laboratory for helpful discussions, Ms. Gretchen Snavely and Ms. Erin Mattern from the Comparative Medicine Histopathology Core, Ms. Jessica Wingate from the Comparative Medicine Diagnostic Laboratory, and Dr. Nataliya Smith, Ms. Kristin Shuler and Mr. John Graybeal from the Department of Neurosurgery’s Neuroscience Research Institute Biorepository for assistance with sample handling and IRB submissions. We are also thankful to the MRI Core Facility staff, Jeffrey Vesek and Patti Miller, for the support on study protocol development/data acquisition/data processing/data analysis in this study and the Microscopy Imaging Core (Leica SP8 Confocal: 1S10OD010756-01A1 CB).

## Conflict of interest

The authors declare that the research was conducted in the absence of any commercial or financial relationships that could be construed as a potential conflict of interest.

## Publisher’s note

All claims expressed in this article are solely those of the authors and do not necessarily represent those of their affiliated organizations, or those of the publisher, the editors and the reviewers. Any product that may be evaluated in this article, or claim that may be made by its manufacturer, is not guaranteed or endorsed by the publisher.
